# Probiotic Bifidobacteria Mitigate the Deleterious Effects of *para*-Cresol in a Drosophila melanogaster Toxicity Model

**DOI:** 10.1128/msphere.00446-22

**Published:** 2022-11-02

**Authors:** Gerrit A. Stuivenberg, John A. Chmiel, Polycronis P. Akouris, Jeremy P. Burton, Gregor Reid

**Affiliations:** a Centre for Human Microbiome and Probiotic Research, Lawson Health Research Institute, London, Ontario, Canada; b Department of Microbiology and Immunology, Western University, London, Ontario, Canada; c Department of Surgery, Western University, London, Ontario, Canada; University of Michigan-Ann Arbor

**Keywords:** chronic kidney disease, host-microbe interactions, probiotics, synbiotics, uremic toxins, *p*-cresol

## Abstract

Renal impairment associated with chronic kidney disease (CKD) causes the buildup of uremic toxins that are deleterious to patient health. Current therapies that manage toxin accumulation in CKD offer an incomplete therapeutic effect against toxins such as *para*-cresol (*p*-cresol) and *p-*cresyl sulfate. Probiotic therapies can exploit the wealth of microbial diversity to reduce toxin accumulation. Using *in vitro* culture techniques, strains of lactobacilli and bifidobacteria from a 24-strain synbiotic were investigated for their ability to remove *p*-cresol. Four strains of bifidobacteria internalized *p*-cresol from the extracellular environment. The oral supplementation of these toxin-clearing probiotics was more protective than control strains in a Drosophila melanogaster toxicity model. Bifidobacterial supplementation was also associated with higher abundance of lactobacilli in the gut microbiota of *p*-cresol-exposed flies. The present findings suggest that these strains might reduce *p-*cresol in the gut in addition to increasing the prevalence of other beneficial bacteria, such as lactobacilli, and should be tested clinically to normalize the dysbiotic gut microbiota observed in CKD patients.

**IMPORTANCE** Chronic kidney disease (CKD) affects approximately 10% of the global population and has limited treatment options. The accumulation of gut microbiota-derived uremic toxins, such as *para*-cresol (*p*-cresol) and *p-*cresyl sulfate, is associated with the onset of comorbidities (i.e., atherosclerosis and cognitive disorders) in CKD. Unfortunately, dialysis, the gold standard therapy is unable to remove these toxins from the bloodstream due to their highly protein-bound nature. Some strains of *Bifidobacterium* have metabolic properties that may be useful in managing uremic toxicity. Using a *Drosophila* model, the present work highlights why dosing with certain probiotic strains may be clinically useful in CKD management.

## INTRODUCTION

Chronic kidney disease (CKD) is a serious condition that affects ~10% of the global population and lacks noninvasive treatment options. Perturbations to kidney function can facilitate the accumulation of toxins by impairing primary solute clearance (1, 2). In late-stage CKD, renal activity is insufficient to manage the continuous production of metabolic waste products; as a result, these compounds persist in the body and intensify disease severity (3, 4). Uremic toxins promote CKD progression by enhancing the production of reactive oxygen species (ROS) and disrupting the normal biological functions of multiple organs (5–8). Renal replacement therapies, such as hemodialysis, have been developed to mitigate the burden of these harmful compounds (9). However, these treatments are expensive and impart an incomplete therapeutic effect, thus highlighting the need for novel strategies that address both the clinical and economic concerns of current regimes (9, 10).

**FIG 1:**
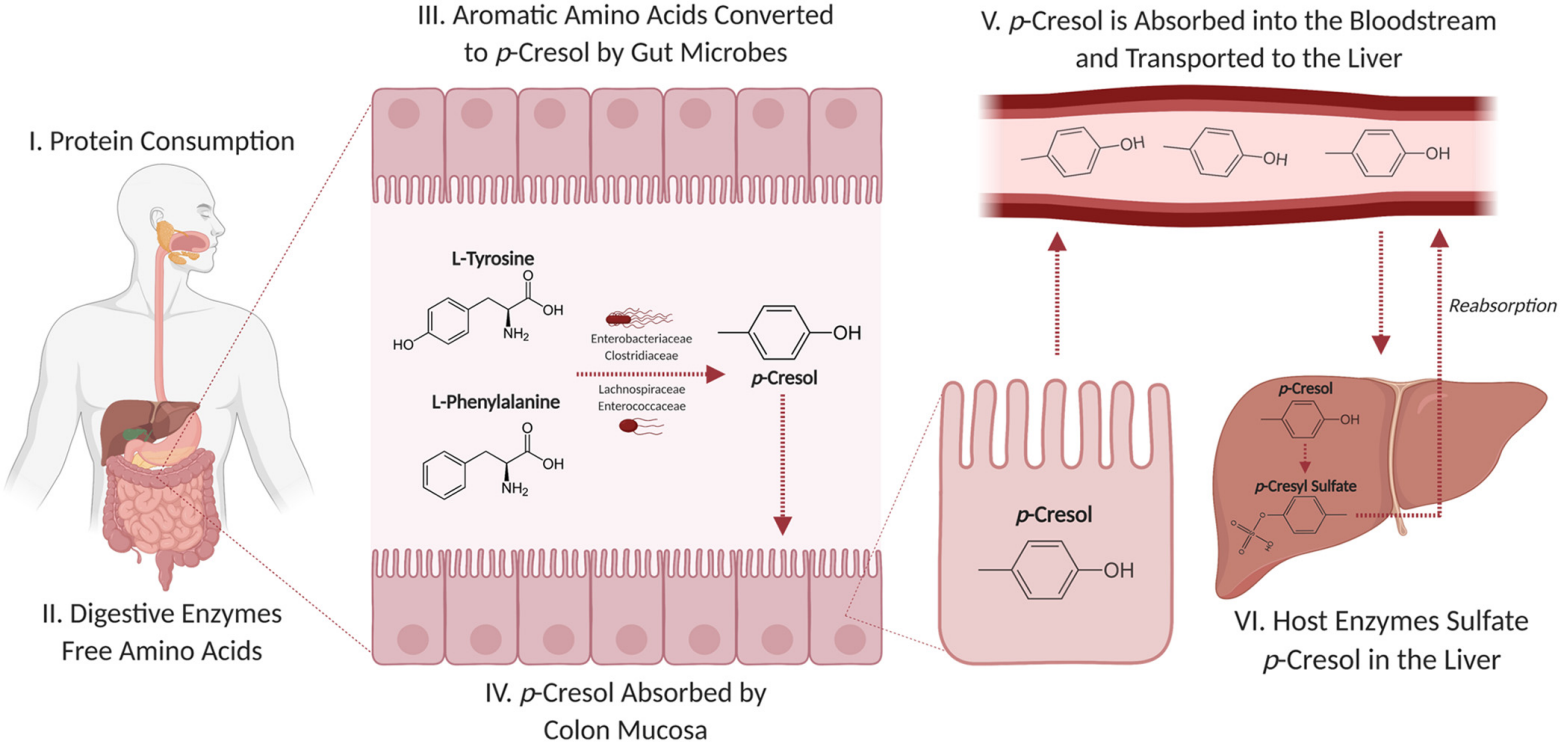
The uremic toxin *p*-cresyl sulfate is sourced from diet-based proteins that are fermented by members of the human gut microbiota. (I) Proteins obtained from the diet are (II) broken down by host enzymes into their constituent amino acids. (III) l-tyrosine and l-phenylalanine are fermented by members of the gut microbiota, forming *p*-cresol, (IV) which is rapidly absorbed by the colon mucosa and (V) transported to the liver, (VI) where it is sulfated into *p*-cresyl sulfate and rapidly binds serum albumin as it enters circulation. Created with BioRender.com.

The buildup of uremic toxins in the body is a risk factor for atherosclerosis in CKD patients (11–13), with one compound, *para*-cresyl (*p*-cresyl) sulfate, being particularly threatening. In addition to accelerating both CKD and atherosclerosis development, *p*-cresyl sulfate exacerbates the risk of cognitive disabilities and other cardiovascular diseases (5, 14–16). *p*-Cresyl sulfate is produced from tyrosine and phenylalanine obtained through the diet (Fig. 1) (17). Briefly, the fermentation of these amino acids by *Clostridium* and *Enterobacteriaceae* spp. residing in the gut produces phenolic compounds, including *p*-cresol (17–19). Tyrosine phenol-lyases and related enzymes liberate phenol groups from their parent amino acid structures for subsequent methylation events that produce *p*-cresol. The resultant compound is then sulfated in the liver and rapidly enters circulation upon binding albumin (20–22).

The retention of uremic waste correlates with functional and compositional changes to the gut microbiota (23, 24). Gastrointestinal secretions of urea into the colonic environment enhance the expression of bacterial enzymes involved in *p-*cresol biosynthesis (25). This is corroborated by data showing that the gut microbiota of end-stage renal disease patients on renal replacement therapy has higher abundance of *Enterobacteriaceae* and *Clostridiaceae* than that of healthy controls (26). Indeed, the gut microbiota of CKD patients is also depleted of lactobacilli and bifidobacteria, which are generally regarded as beneficial and frequently appear in probiotic formulations (26–29). These data highlight the potential of gut-targeted approaches to remedy the microbial dysbiosis and uremic toxicity associated with renal decline.

Multiple studies have assessed probiotic administration to quell uremic toxicity with various degrees of success. Oral supplementation of mixtures containing strains of either Bifidobacterium breve or Bifidobacterium longum can decrease serum *p-*cresol by normalizing the intestinal microbiota and subsequently slowing CKD progression (30, 31). However, strain-specific contributions from these studies are difficult to discern because the probiotic formulations contained prebiotics, which can stimulate certain genera. Although the observed reduction of *p*-cresol/*p*-cresyl sulfate was assumed to be a result of a more balanced intestinal microbiota, there is no definition of a true “normal” microbiota, and this overlooks the potential of these bacteria to directly interact with toxins in the environment.

In the present study, the hypothesis that some probiotic bifidobacteria and lactobacilli can sequester free *p*-cresol was tested. Using a combination of *in vitro* techniques and a Drosophila melanogaster model, we sought to determine if individual strains isolated from a commercial synbiotic (Seed Health, CA, USA) attenuate uremic toxicity through direct interactions with *p*-cresol.

## RESULTS

### Four strains of *Bifidobacterium* sequester *p*-cresol *in vitro*.

**FIG 2:**
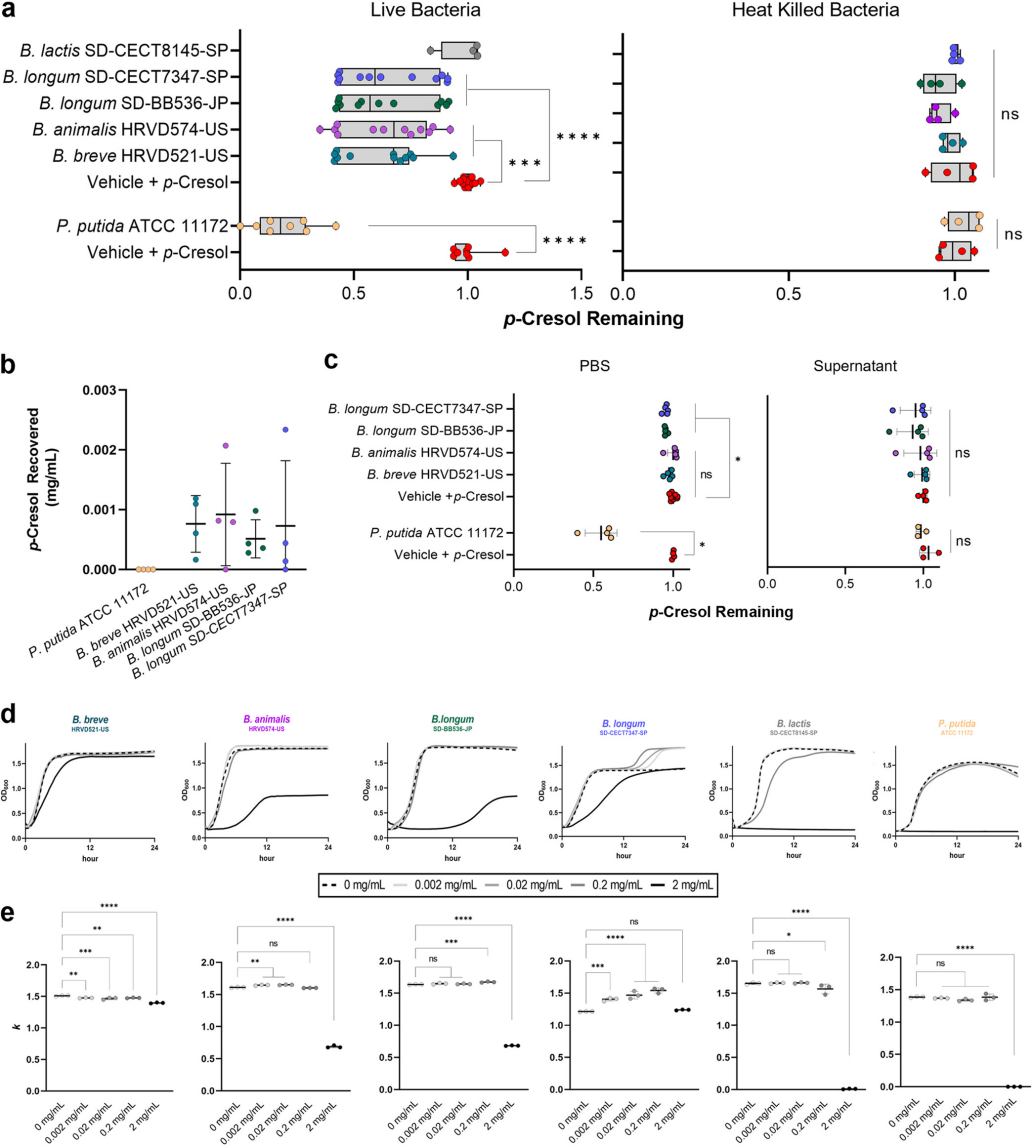
Environmental *p-*cresol is depleted by probiotic *Bifidobacterium* sp. strains and enhances their growth *in vitro.* (a) Live or heat-killed *Bifidobacterium* sp. strains and P. putida ATCC 11172 (*n* = 4 to 12) were grown in minMRS or minNB with *p*-cresol at 0.2 mg/mL, respectively. *p*-Cresol remaining in the supernatant was quantified after 24 h of growth. (b) Following incubation with the toxin, bacterial cells were washed and lysed to assess *p*-cresol content. (c) Bacteria were cultured for 24 h in the appropriate medium, and cells were removed prior to spiking spent supernatant with 0.2 mg/mL *p*-cresol (*n* = 3 or 4). The removed cells were washed and added to PBS with *p*-cresol at 0.2 mg/mL for 24 h (*n* = 4 or 5). In the graphs, data are displayed as the mean ± standard deviation (SD). (d and e) The ability to persist in high concentrations of *p*-cresol (d) was assessed by culturing strains in increasing concentrations of the toxin (*n* = 3) and determining the subsequent (e) carrying capacity (*k*). Growth is represented as the change in optical density at 600 nm over 24 h. In the box plot diagrams, boxes represent the first and third quartile values, while black lines denote medians. Whiskers encompass maximum and minimum values. All other data are displayed as the mean ± SD. Significance was determined by the Mann-Whitney test or Kruskal-Wallis test; *, *P < *0.05; **, *P < *0.01; ***, *P < *0.001; ****, *P < *0.0001; ns, not significant.

To identify lactic acid bacteria that sequester uremic toxins, individual probiotic strains from the commercial product (Table 1) were cultivated in the presence of *p-*cresol. A blinded screen of 24 strains indicated that 4 reduced the toxin *in vitro* (see Fig. S1 in the supplemental material). Replication, including a *p*-cresol-degrading control bacterium, confirmed a significant difference in *p*-cresol content between group medians (Fig. 2a; Kruskal-Wallis; H = 43.74, *P* < 0.0001). Specifically, B. breve HRVD521-US (*P* = 0.0001), Bifidobacterium animalis HRVD574-US (*P* = 0.0004), B. longum SD-BB536-JP (*P* = 0.0009), and B. longum SD-CECT7347-SP (*P* = 0.0016) lowered the amount of *p-*cresol after 24 h of growth compared to the uninoculated control (Fig. 2; Dunn’s multiple comparison; α = 0.05) despite a high degree of intrastrain variation. *B. lactis* SD-CECT8145-SP did not diminish *p-*cresol (*P* > 0.9999). As expected, the control strain, Pseudomonas putida ATCC 11172, also reduced *p-*cresol from culture (Fig. 2a; Mann-Whitney test; *P* = 0.0002). No probiotic strain could clear the toxin more efficiently than P. putida ATCC 11172, a phenol-degrading pseudomonad (32). The ability to reduce *p*-cresol was lost in the toxin-clearing probiotic strains (Fig. 2a; one-way analysis of variance [ANOVA]; *F* = 1.229, *P* = 0.3402) and P. putida ATCC 111 (Fig. 2a: two-tailed *t* test; *P* = 0.4015) following heat inactivation.

**TABLE 1:** Composition of Seed synbiotic^*a*^

Species	Strain(s)
Bifidobacterium adolescentis	SD-BA5-IT (DSM18352)
Bifidobacterium breve	SD-BR3-IT HRVD521-US
Bifidobacterium infantis	SD-M63-JP
Bifidobacterium lactis	HRVD524-US (Bl-04)
Bifidobacterium longum	SD-BB536-JP HRVD90b-US SD-CECT7347-SP
Bifidobacterium lactis	SD-BS5-IT SD150-BE SD-CECT8145-SP SD-MB2409-IT
Lacticaseibacillus casei	HRVD300-US SD-CECT9104-SP
Lacticaseibacillus rhamnosus	HRVD113-US SD-GG-BESD-LR6-IT
Lactiplantibacillus plantarum	SD-LPLDL-UK SD-LP1-IT
Ligilactobacillus salivarius	SD-LS1-IT
Limosilactobacillus fermentum	SD-LF8-IT
Limosilactobacillus reuteri	RD830-FR SD-LRE2-IT

a The product contains microbiota-accessible prebiotics, 400 mg: polyphenol pomegranate (whole fruit and skin with 30% punicalagins), organic pine bark flavonoids (50% oligomeric proanthocyanidins), and organic chaga mushroom polysaccharides.

10.1128/msphere.00446-22.1 FIG S1 Blind screen for *p*-cresol clearing ability in 23 lactic acid bacteria isolated from a commercial probiotic product. Download FIG S1, PDF file, 0.1 MB. Copyright © 2022 Stuivenberg et al. 2022 Stuivenberg et al. https://creativecommons.org/licenses/by/4.0/ This content is distributed under the terms of the Creative Commons Attribution 4.0 International license.

Following *p*-cresol uptake, an attempt was made to recover the toxin from whole-cell lysate of exposed bacteria. Although the toxin was not fully recovered, *p*-cresol was retained in the cell pellets of the *Bifidobacterium* strains (Fig. 2b) but not P. putida ATCC 11172. Although the median *p-*cresol recovery was not significantly different between probiotic groups and P. putida (Fig. 2b; Kruskal-Wallis; H = 7.970, *P* = 0.0927), Dunn’s multiple comparison revealed that significantly more *p-*cresol was recovered from B. breve HRVD521-US (*P* = 0.0439).

For more insight on how *p*-cresol is sequestered by probiotic strains, clearance was assessed under additional conditions. Filter-sterilized supernatant of bacterial cultures was insufficient in removing the *p*-cresol spiked into solution for the four toxin-clearing probiotics (Fig. 2c; Kruskal-Wallis; H = 6.414, *P* = 0.1703) and the P. putida strains (Fig. 2c; Mann-Whitney test, *P* = 0.400). Furthermore, when live cells were limited metabolically by being suspended in 1× phosphate-buffered saline (PBS) spiked with *p*-cresol, only B. longum SD-BB536-JP (*P* = 0.0147), B. longum SD-CECT7347-SP (*P* = 0.0218), and P. putida ATCC 11172 (*P* = 0.0286) significantly lowered *p*-cresol in the solution (Fig. 2c).

### *p*-Cresol enhances the growth of some toxin-clearing bifidobacteria.

Since *p-*cresol is toxic to microorganisms, the viability of the probiotic strains was assessed in *p-*cresol concentrations ranging from 0.002 mg/mL to 2 mg/mL. The results highlight the differential effect that *p*-cresol has on the growth of each strain (Fig. 2d  and e). In the presence of *p*-cresol at concentrations of ≤0.2 mg/mL, B. longum SD-CECT7347-SP received a distinct and significant growth advantage (Fig. 2e; one-way ANOVA; *F* = 52.37, *P* < 0.0001) compared to the vehicle control at all concentrations below 2 mg/mL (Dunnett’s multiple comparison; *P* > 0.0002). *B. animalis* HRVD574-US (Fig. 2e; one-way ANOVA; F = 5681, *P* < 0.0001) and B. longum SD-BB536 (one-way ANOVA; F = 10399, *P* > 0.0001) also grew better at 0.002 and 0.02 mg/mL (Dunnett’s multiple comparison; *P* < 0.01) or 0.2 mg/mL (*P* < 0.001), respectively, compared to the nonspiked controls. Alternatively, B. breve HRVD521-US (Fig. 2e; one-way ANOVA; *F* = 55.96; *P* < 0.0001) and *B. lactis* SD-CECT8145-SP (one-way ANOVA; *F* = 1,261; *P* < 0.0001) received no growth advantage in the presence of *p*-cresol and were instead negatively impacted by the presence of toxin at nearly every concentration (Fig. 2d and e). Surprisingly, P. putida ATCC 11172 also did not benefit from *p*-cresol in culture despite the ability to use it as a carbon source and demonstrated restricted growth at 2 mg/mL (Fig. 2e; one-way ANOVA; *F* = 1862; *P* < 0.0001). The growth of all strains was negatively impacted when exposed to 2 mg/mL of *p*-cresol compared to the nonspiked controls (Dunnett’s multiple comparison; *P* < 0.0001). However, each toxin-clearing probiotic strain faired better at the highest concentration of *p-*cresol than the controls P. putida ATCC 11172 and *B. lactis* SD-CECT8145-SP.

### Probiotic bifidobacteria do not produce *p*-cresol.

**FIG 3:**
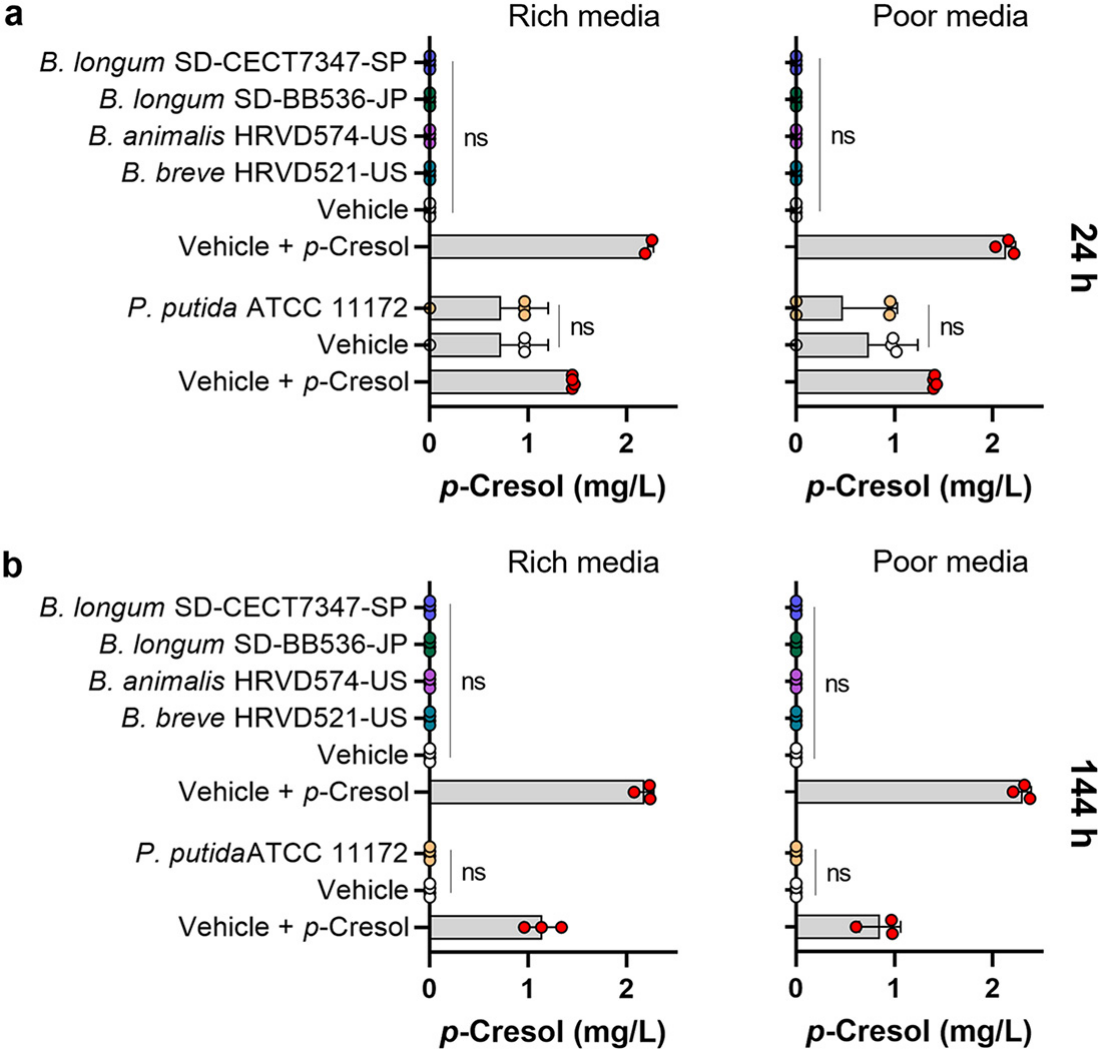
Toxin-clearing bifidobacteria do not produce *p*-cresol. (a and b) The five toxin-clearing bacterial strains were cultured in rich or poor medium for (a) 24 to (b) 144 h. The concentration of *p*-cresol produced is displayed as the mean ± SD (*n* = 3 or 4). Significance was determined by comparing bacterial cultures to uncultured control medium with the Mann-Whitney test or Kruskal-Wallis test; *, *P < *0.05; **, *P < *0.01; ***, *P < *0.001; ****, *P < *0.0001; ns, not significant.

Because some *Bifidobacterium* spp. have been reported to produce *p*-cresol (19), the four strains of interest were tested for this. Compared to the no-bacteria controls, neither the probiotic strains (Fig. 3a and b; Kruskal-Wallis; *P* > 0.05) nor P. putida ATCC 11172 (Fig. 3a and b; Mann-Whitney test; *P* > 0.05) significantly altered *p*-cresol content.

### *p*-Cresol internalizing bifidobacteria extend the life span of Drosophila melanogaster in a *p*-cresol toxicity model.

The potential of the probiotic strains to reduce *p*-cresol toxicity *in vivo* was evaluated in D. melanogaster. To confirm previous findings that *p*-cresol is deleterious to some mutant *Drosophila* (33), the effect of oral *p*-cresol exposure on the fly life span was determined. Using the capillary feeder (CAFE) assay, *p*-cresol exposure did not affect WT D. melanogaster Canton-S flies (Fig. S2a; log-rank [Mantel-Cox]; chi-squared = 3.939; *df* = 2; *P* = 0.1395); D. melanogaster w^1118^ exposed to *p*-cresol in this manner (≥1 mg/mL) demonstrated a significant reduction (Fig. S2b; log-rank [Mantel-Cox]; chi-squared = 42.16 [1 mg/mL], 49.98 [5 mg/mL], 53.99 [10 mg/mL], 85.80 [15 mg/mL]; *df* = 1; *P* < 0.0001) in survival. *p*-Cresol supplemented to D. melanogaster w^1118^ flies in standard rearing media also significantly reduced the life span (Fig. S2c; log-rank [Mantel-Cox]; chi-squared = 297.7; *df* = 2; *P* < 0.0001); however, complete lethality was achieved too quickly to facilitate experimentation on a longer timescale. Thus, the following experiments used D. melanogaster w^1118^ flies exposed to 10 mg/mL *p*-cresol using the CAFE assay (34).

10.1128/msphere.00446-22.2 FIG S2 *p*-Cresol reduces the lifespan of *w^1118^* flies. Download FIG S2, PDF file, 0.2 MB. Copyright © 2022 Stuivenberg et al. 2022 Stuivenberg et al. https://creativecommons.org/licenses/by/4.0/ This content is distributed under the terms of the Creative Commons Attribution 4.0 International license.

**FIG 4:**
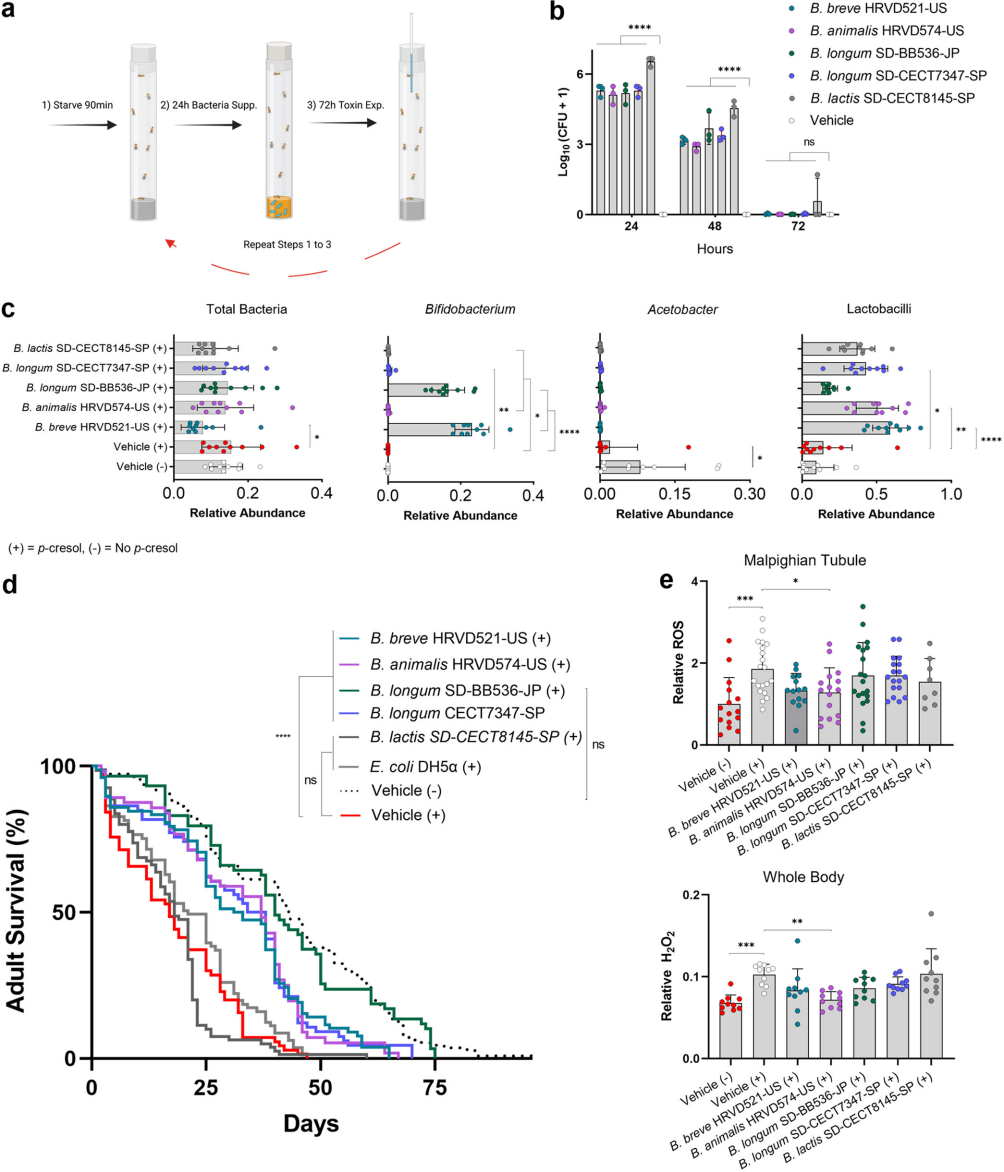
Toxin-clearing *Bifidobacterium* sp. strains can colonize *Drosophila* and extend the life span of *p*-cresol-exposed flies. (a) Summary of experimental design; prior to *p*-cresol exposure, flies were starved and supplemented with one of four toxin-clearing *Bifidobacterium* strains or controls. Created with BioRender.com. (b) CFU of *Bifidobacterium* spp. per fly plated on *Bifidobacterium* specific agar (*n* = 3). Data are displayed as the geometric mean of log_10_(CFU + 1) ± SD. (c) qPCR-based quantification of dominant genera and supplemental bifidobacteria across treatment groups after 1 week of toxin exposure and bacterial supplementation. (d) Survival curves for *w^1118^* flies on *p*-cresol (10 mg/mL) or vehicle (5% [wt/vol] sucrose in ddH_2_O) and recurrent bacterial supplementation (*n *=* *20 to 30 from 2 to 3 independent replicates for each treatment group). (e) ROS was quantified in the Malpighian tubule and whole bodies of flies after 1 week of toxin exposure and bacterial supplementation. Unless otherwise stated, data are displayed as the mean ± SD. Statistical analyses shown are from one-way ANOVA, Kruskal-Wallis, or log-rank (Mantel-Cox) tests. *, *P < *0.05; **, *P < *0.01; ***, *P < *0.001; ****, *P < *0.0001; ns, not significant.

Since bifidobacteria are not native to *Drosophila*, verifying that the four strains could colonize D. melanogaster w^1118^ flies was imperative. The toxin-clearing and control *Bifidobacterium* strains were detected by culture on bifidobacterium-specific agar (BSA) (35) from homogenized flies after 72 h. Bifidobacterial loads were significantly higher than controls at 24 h (Fig. 4b; one-way ANOVA; *F* = 157.6; *P* < 0.0001) and 48 h (one-way ANOVA; *F* = 34.89; *P* < 0.0001) postsupplementation. However, after 72 h, no difference in the amount of culturable bifidobacteria could be detected across all treatment groups (one-way ANOVA; *F* = 1.310; *P* = 0.3232).

To investigate if the four *Bifidobacterium* strains could recover life span and modulate the microbiota of *p*-cresol-exposed flies, an experimental design that considers the retention of the strains is highlighted in Fig. 4a. Compared to the no-bacteria controls, B. breve HRVD521-US (chi-squared = 29.48), *B. animalis* HRVD574-US (chi-squared = 27.30), B. longum SD-BB536-JP (chi-squared = 53.70), and B. longum SD-CECT7347-SP (chi-squared = 28.22) significantly extended the D. melanogaster w^1118^ life span (Fig. 4d; log-rank [Mantel-Cox]; *df* = 1; *P* < 0.0001). While a complete recovery of life span was unexpected, B. longum SD-BB536-JP restored the life span such that it was not significantly different from the no-*p*-cresol controls (Fig. 4d; log-rank [Mantel-Cox]; chi-squared = 0.04458; *df* = 1; *P* = 0.8328). Escherichia coli DH5α and *B. lactis* SD-CECT8145-SP, which cannot clear *p*-cresol, had no effect on D. melanogaster w^1118^ life span compared to nonsupplemented *p*-cresol-exposed control flies. The presence of toxin in the sucrose solution did not seem to alter palatability (Table S1).

10.1128/msphere.00446-22.3 TABLE S1 Capillary feeder assay food consumption measurements. Download Table S1, XLSX file, 0.03 MB. Copyright © 2022 Stuivenberg et al. 2022 Stuivenberg et al. https://creativecommons.org/licenses/by/4.0/ This content is distributed under the terms of the Creative Commons Attribution 4.0 International license.

### *p*-Cresol exposure and bifidobacterial supplementation alter the microbial profile of D. melanogaster w^1118^.

Quantitative PCR was used to determine the change in bacterial load in response to *p*-cresol exposure and probiotic supplementation. A difference in abundance of total bacteria between all treatment groups was reported (Fig. 4c; Kruskal-Wallis; H = 15.06; *P* = 0.0198), but Dunn’s multiple comparison revealed that only B. breve HRVD521-US had fewer bacteria than the nonsupplemented control group that received *p*-cresol (*P* = 0.0135). Interestingly, toxin exposure altered the abundance of *Acetobacter* (Fig. 4c; Kruskal-Wallis; H = 28.41; *P* < 0.05); All treatment groups had significantly less *Acetobacter* than flies that did not take in *p*-cresol (Dunn’s multiple comparison; *P* < 0.05). Despite CFU enumeration suggesting a lack of bifidobacteria 72 h after supplementation (Fig. 4b), the abundance of *Bifidobacterium* spp. was significantly higher in each probiotic-treated group than in controls (Kruskal-Wallis; H = 61.90; *P* < 0.0001); however, B. longum SD-BB536-US and B. breve HRVD521-US appeared to be the most efficient colonizers of *Drosophila*, as they were present in much higher quantities (Dunn’s multiple comparison; *P* < 0.0001). Bifidobacterial supplementation was also associated with more lactobacilli present in the Drosophila melanogaster gut.

### B. longum HRVD574-US reduces oxidative stress in *p*-cresol-exposed Drosophila melanogaster w^1118^.

To determine if bacterial supplementation could reduce the burden of ROS resulting from *p*-cresol exposure, D. melanogaster Malpighian tubules (i.e., fly kidneys) were stained and imaged with a confocal microscope. ROS were detected in the Malpighian tubules of all flies across different treatment groups (Fig. 4e). Quantification revealed a significant difference in the relative fluorescence of the Malpighian tubules between treatment groups (one-way ANOVA; *F* = 3.927; *P* = 0.0014). Dunnett’s multiple comparison showed that *p*-cresol-exposed flies had more ROS than the unexposed controls (*P* = 0.0005); flies that were supplemented with B. breve HRVD574-US and *p*-cresol had fewer ROS than the nonsupplemented, toxin exposed controls (*P* = 0.0343).

As an additional measure of oxidative stress, the burden of H_2_O_2_ in whole flies was also assessed. Quantification revealed a significant difference in H_2_O_2_ between treatment groups (Fig. 4e, one-way ANOVA; *F* = 5.964; *P* < 0.0001). According to Dunnett’s multiple comparisons, *p*-cresol-exposed flies had a higher H_2_O_2_ content than unexposed controls (*P* < 0.001). Despite each toxin-clearing bifidobacterium lowering the amount of H_2_O_2_ relative to the uninoculated control, this observation was only significant for B. longum HRVD574-US (*P* < 0.01).

## DISCUSSION

This study is the first to identify that *Bifidobacterium* strains reduce *p-*cresol *in vitro* and mitigate its toxicity in *Drosophila*. Toxin clearance relied on live whole cells under conditions that facilitated normal metabolic activity. Remarkably, when the *Bifidobacterium* strains were supplemented to *p*-cresol-exposed flies, a recovery of life span and changes to the fly microbiota were observed. These findings highlight the potential of selected bifidobacterial strains to remove *p*-cresol *in vitro* and mitigate *p*-cresol toxicity *in vivo*, thus supporting their clinical application with greater mechanistic insight than previous studies.

It has already been proposed that reducing *p*-cresol availability in the gut might delay CKD progression and the onset of comorbidities (5, 6, 12). This has resulted in gut-targeted therapies that require daily consumption of ~30 capsules of an oral phenol absorbent (36). However, the consumption of 1 to 2 probiotic capsules would be less burdensome, and the findings here suggest they could have clinical utility. The present study reports the largest reduction of *p*-cresol by any probiotic microbe, with B. longum SD-BB536-JP, B. breve HRVD521-US, *B. animalis* HRVD574-US, and B. longum SD-CECT7347 clearing approximately 30 to 40% of *p*-cresol *in vitro*. Only B. longum SD-BB536-JP was expected to reduce the toxin, because it has been shown to slow CKD progression and reduce serum *p*-cresol/*p*-cresyl sulfate in humans and rats (30, 31). The ability of these strains to reduce *p*-cresol after being isolated from an over-the-counter synbiotic indicates that the organisms retained their functionality following commercial processing. While the original product contains 24 strains, including lactobacilli that do not utilize *p*-cresol, it is worth clinical testing of the entire product because the gut microbiota of CKD patients is depleted of both *Bifidobacterium* and *Lactobacillus* spp. (26). In addition to potentially reducing *p*-cresol, supplementing both genera to these individuals could provide an additive effect by countering dysbiosis associated with the disease (26, 37).

Even though *p*-cresol is produced and degraded by bacteria, the compound confers toxicity to human commensals by disrupting cellular membranes (38–40). Here, the toxin-clearing *Bifidobacterium* strains demonstrated either normal or enhanced proliferative capacity in the presence of *p*-cresol, exceeding physiological extremes (41); the dramatic increase in carrying capacity of B. longum CECT7347-SP in the presence of *p*-cresol suggests it may be used as a nutrient source. The ability to clear *p*-cresol by probiotics likely confers resistance against the compound because the growth of the control strain, *B. lactis* SD-CECT8145-SP, was negatively affected at almost every concentration and could not survive at 2 mg/mL. Additionally, the four toxin-clearing strains persisted for a few days in the suboptimal intestinal environment defined by the D. melanogaster gut (42, 43). Although humans are a more suitable host for bifidobacteria, retention of these strains by *Drosophila* was expected because it has been done previously (44–46). A recent study also confirmed the ability of these probiotic strains to survive the various conditions found in the human gastrointestinal tract (47). Taken together, these results indicate that the four *p*-cresol-clearing bifidobacteria will thrive in the intestinal environment of CKD patients even at extremes of *p-*cresol/*p*-cresyl sulfate-associated uremic toxicity.

Although *p*-cresol was found inside bifidobacterial cells, its fate upon internalization is unclear and currently being elucidated. Due to the observed increase in growth, it is likely that the probiotics can use the toxin as a nutrient source; however, no *p*-cresol-catabolizing enzymes have been characterized in bifidobacteria. P. putida ATCC 11172 can utilize *p*-cresol as its sole carbon source and greatly reduced the compound in PBS (32, 48). The lack of this ability in two of the four bifidobacteria tested suggests that *p*-cresol is used differently among the strains. Notably, the ability to degrade *p*-cresol is likely not necessary for clinical efficacy. Since orally supplemented probiotics are transient in the host gut (49), it is suspected that passage of these strains through the gastrointestinal tract will increase *p*-cresol excreted in the feces via their cellular internalization of the compound. Although some *Bifidobacterium* strains have been shown to produce *p*-cresol at the species level (19), the strains in this study do not, further supporting their clinical application. Since the effect of probiotics are strain specific (50), it is easy to speculate that the *p*-cresol-clearing *Bifidobacterium* strains could have adapted cellular machinery used in phenol production for other functions, such as toxin clearance. The mechanistic details behind *p*-cresol uptake are already being explored by our group.

Presently, there is no *in vivo* model that facilitates the easy testing of gut-targeted therapies to manage uremic toxicity. *Drosophila* are well characterized for their use as a high-throughput model for host-microbiota interactions (45, 51). O’Dell and colleagues showed that high concentrations of *p*-cresol killed nearly half of the exposed *w^1118^* flies in just 10 h (33), and these findings were confirmed here. The present study also proved that the daily consumption of low concentrations of *p*-cresol spiked into sucrose solution is toxic to *w^1118^* flies. The observation that *w^1118^* but not wild-type (WT) flies are susceptible to *p*-cresol could be explained by their phenotypic aberrations (52). Octopamine is an important hormone in *Drosophila* physiology that can offer protection from *p*-cresol (33, 53). Compared to the WT, *w^1118^* flies have a reduced capacity to circulate such hormones, and this might mitigate their protective properties (52, 54, 55). In line with these findings, *w^1118^* flies have a dampened stress tolerance and are more susceptible to compounds such as pesticides and ROS than WT flies (52, 56). In humans, *p*-cresol enhances ROS production (57, 58). The observed increase of ROS in both the whole body and Malpighian tubules of *w^1118^* flies following *p*-cresol exposure suggests the toxin effects these organisms in a similar manner and further validates this model.

Human probiotic strains can enhance *Drosophila* longevity and confer tolerance to a wide variety of stressors (44, 45). Thus, the four toxin-clearing *Bifidobacterium* strains were expected to improve the life span of *p*-cresol-exposed flies. In compliance with these expectations, B. breve HRVD521-US, *B. animalis* HRVD574-US, and B. longum SD-CECT7347-SP improved *w^1118^* tolerance to *p*-cresol as evidenced by an approximately 20-day increase in life span. Bacterial supplementation alone was insufficient to extend life span, as neither the nondegrader *B. lactis* SD-CECT8145-SP nor E. coli DH5α had any effect on life span. For *B. animalis* HRVD574-US, the increase in longevity may be partly explained by preventing the production of ROS after *p*-cresol exposure. This is notable because some *Bifidobacterium* spp. increase ROS in *Drosophila* (59). In CKD patients, limiting ROS production could prevent the emergence of comorbidities such as atherosclerosis (60). It was surprising that B. longum SD-BB536-JP completely ameliorated *p*-cresol toxicity without reducing ROS; however, the reason behind this observation is not yet clear.

In CKD, *p*-cresol reduces short-chain fatty acid-producing bacteria residing in the gut (61–63). A similar effect was shown here in *Drosophila*, where *p-*cresol exposure reduced the abundance of *Acetobacter*. *Drosophila* rely heavily on acetate produced by *Acetobacter* to regulate growth and development (64–66). Interestingly, bifidobacterial supplementation regardless of *p*-cresol clearing ability increased the abundance lactobacilli in these flies. It is easy to postulate that reestablishing acetate and other short-chain fatty acid-producing bacteria, such as bifidobacteria and lactobacilli, might promote normal development under toxin stress (67). However, despite *B. lactis* SD-CECT8145-SP yielding the same changes to the fly microbiota, only the toxin-clearing strains protected against *p*-cresol. This highlights the importance of strain selection and suggests that enhanced longevity in D. melanogaster did, in part, rely on the ability to clear *p*-cresol.

While it is promising that *p*-cresol impacted *Drosophila* similarly to humans, as evidenced by increased ROS and microbial shifts (57, 58, 62, 63), there are some major shortcomings that must be considered. The simplified microbiota, reliance on innate immunity, and differences in GI physiology may hamper the translatability of findings from this model (43, 68, 69). But the fact that several *Bifidobacterium* strains, including some used in this study, have already been proven clinically useful against CKD indicates that the translatability of our findings may not be a concern (30, 31).

In conclusion, this study showed that four *Bifidobacterium* sp. strains sequester *p*-cresol in broth culture and reduced its toxicity in *Drosophila*. Our work highlights the potential of these probiotics to be a therapeutic adjunct in managing uremic toxicity in CKD and offers new insight to how this may be achieved.

## MATERIALS AND METHODS

### Chemicals.

*p*-Cresol (C040025G), 3-(*p*-hydroxyphenyl) propionate (M21965G), and 4-hydroxyphenylacetate (H029025G) were obtained from Fisher Scientific. 4-Hydroxyphenylpyruvate (114286-1G) and dl-4-hydroxyphenyllactate (H3253-100mg) were obtained from Sigma. 4-Hydroxybenzoate (AAAB24016-14) was obtained from VWR. All chemicals were stored under appropriate conditions as defined by the manufacturer. Appropriate concentrations were made the day of experimental use in acetonitrile (ACN; Fisher, A996-4) or 5% [wt/vol] sucrose in double-distilled water (ddH_2_O).

### Overnight culture preparation.

All strains from a 24-strain synbiotic were received in monoculture and verified with Gram-strain. Probiotic strains (Seed synbiotic) were streak-plated from frozen stock onto De Man, Rogosa, and Sharpe (MRS) solid medium (BD Difco) containing 0.5 g/L l-cysteine (Sigma) and incubated anaerobically (BD GasPak catalog no. 260678) at 37°C under stationary conditions for 24 h. Single colonies were selected and inoculated overnight at 37°C in MRS (with 0.5 g/L l-cysteine) broth anaerobically and under stationary conditions to produce experimental cultures.

Pseudomonas putida ATCC 11172 was streak-plated from frozen stock onto nutrient broth (5 g peptone and 3 g beef extract dissolved in 1 L ddH_2_O) agar and incubated aerobically at 30°C under stationary conditions overnight. A single colony was selected and inoculated aerobically overnight at 30°C in nutrient broth with shaking (200 rpm) to produce experimental cultures.

Escherichia coli DH5α was streak-plated from frozen stock onto LB agar and incubated aerobically at 37°C under stationary conditions overnight. A single colony was selected and inoculated aerobically overnight at 37°C in LB broth under stationary conditions.

### Clearance of *p*-cresol *in vitro*.

Probiotic strains were subcultured (1:50) into a minimal MRS (minMRS) broth with a reduced carbon source (0.50 g l-cysteine HCl, 5 g dextrose, 10 g protease peptone no. 3, 5.0 g yeast extract, 2.0 g ammonium citrate, 5.0 g sodium acetate, 0.1 g magnesium sulfate·7H_2_O, 0.05 g manganese sulfate·H_2_O, 1 mL polysorbate 80, 11 mL potassium phosphate dibasic [stock 1 M], 3 mL iron sulfate [stock 0.1 M], dissolved in 1 L ddH_2_O), and P. putida ATCC 11172 was subcultured into nutrient broth diluted (1:2) with ddH_2_O. Each tube was spiked with 0.2 mg/mL *p*-cresol. The concentration of *p*-cresol used mimics physiological extremes of the human intestinal tract (41). The probiotic strains were incubated anaerobically for 24 h at 37°C under stationary conditions, and P. putida ATCC 11172 was incubated aerobically for 24 h at 30°C with shaking (200 rpm) prior to high-pressure liquid chromatography (HPLC) analysis; P. putida ATCC 11172 was used as a positive control because it is known to degrade *p*-cresol (32). *p*-Cresol-clearing strains were subject to further replication using the same protocol.

### Determining *p*-cresol tolerance *in vitro*.

Overnight cultures were subcultured (1:100) into 96-well plates (Falcon catalog no. 35177) with appropriate media and either 0 mg/mL, 0.002 mg/mL, 0.02 mg/mL, 0.2 mg/mL, or 2 mg/mL of *p*-cresol. Plates were incubated under the appropriate conditions for 24 h, and the optical density was measured every 30 min at 600 nm using a microplate reader (BioTek, Eon). Growth curves were analyzed using the R package Growthcurver v0.3.1with the “min” background correction method (70).

### Clearance of *p*-cresol in PBS.

Overnight cultures were centrifuged (4,500 × *g* for 10 min at ambient temperature), and the cell pellets were washed thrice with sterile 1× phosphate-buffered saline (PBS). The washed cells were transferred to fresh PBS spiked with 0.2 mg/mL *p*-cresol (71) and were incubated under the appropriate conditions for 24 h prior to HPLC analysis.

### Clearance of *p*-cresol in spent culture supernatant.

Overnight cultures were centrifuged (4,500 × *g* for 10 min at ambient temperature), and the supernatant was decanted. The supernatant was filter-sterilized with a 0.22-micron filter (FroggaBio, SFO.22PES), and 0.2 mg/mL of *p*-cresol was added. The samples were then incubated under the appropriate conditions for 24 h prior to HPLC analysis.

### Clearance of *p-*cresol by heat-killed bacteria.

Overnight cultures were centrifuged (4,500 × *g* for 10 min at ambient temperature), and the supernatant was discarded. The pellet was washed once and then resuspended in 1 mL PBS. Inactivation was achieved by incubating the samples on a 100°C heat block for 10 min. The heat-killed cells were resuspended in 10 mL of the relevant medium spiked with 0.2 mg/mL *p*-cresol and were grown overnight under the appropriate conditions prior to HPLC analysis. Efficient inactivation was ensured by plating the bacteria on the appropriate solid medium.

### Recovering *p-*cresol from the cell pellet.

Overnight cultures were subcultured (1:50) into the appropriate minimal medium containing *p*-cresol. Samples were incubated for 24 h under the appropriate conditions. Following incubation, the cells were washed twice with PBS and lysed prior to HPLC analysis.

### Assessing *p*-cresol production.

Strains of interest were screened for *p*-cresol production using a method described by Saito et al. (19). Briefly, overnight cultures were individually subcultured (1:50) into the appropriate culture medium (rich medium: MRS or TSB; poor medium: Peptone Yeast Glucose Broth (PYG) or dilute Nutrient Broth (NB)) containing 200 μM (each) tyrosine and its predicted microbial metabolic intermediates, including 4-hydroxyphenylpyruvate, dl-4-hydroxyphenyllactate, 3-(p-hydroxyphenyl) propionate, 4-hydroxyphenylacetate, and 4-hydroxybenzoate, and incubated under appropriate conditions for 24 to 144 h prior to HPLC analysis (19).

### Sample preparation and HPLC conditions.

All samples for HPLC analysis were first centrifuged at 4,500 × *g* for 10 min at ambient temperature. The supernatant was collected and mixed 1:1 with acetonitrile before resting at 4°C for 10 min. Samples were then centrifuged at 13,000 × *g* for 10 min at 4°C, and the resulting supernatant was filtered (0.22 μm) into light-protected HPLC vials. Samples were stored at 4°C for no more than 48 h prior to analysis.

All samples and standards were analyzed with an Agilent 1100 HPLC device equipped with a degasser (G1379A), quaternary pump (G1311A), autosampler (G1313A), and diode array detector (G1315B). All analyses were performed on an Agilent Poroshell 120 EC-C_18_ (4.6- by 150-mm inside diameter [i.d.], 4-μm particle size) column at ambient temperature. All acetonitrile (Fisher catalog no. A996-4) and water (Fisher catalog no. W5-4) used were HPLC grade. The mobile phase consisted of an isocratic mixture of acetonitrile plus 0.1% orthophosphoric acid and water plus 0.1% orthophosphoric acid (40:60 [vol/vol]) at a flow rate of 0.8 mL/min. The sample injection volume was 20 μL, and detection was performed at 222 nm. Run times were 7 min, with *p*-cresol eluting at ~5 min. Data were analyzed using ChemStation A.10.02. The peak area of samples was compared with the peak area of the external calibration curve (0.001 to 0.2 mg/mL) to quantify *p*-cresol. Standards were prepared in a 1:1 mixture of acetonitrile and water.

### Drosophila melanogaster husbandry.

Drosophila melanogaster Canton-S (RRID: BDSC_1) and white mutant *w^1118^* (RRID: BDSC_3605) flies were obtained from the Bloomington Drosophila Stock Center (NIH P40ODO18537) at Indiana University. All flies were maintained using media with 1.5% (wt/vol) agar, 1.73% (wt/vol) yeast (Sigma-Aldrich catalog no. 51475), 7.3% (wt/vol) cornmeal, 7.6% (vol/vol) corn syrup, and 0.58% (vol/vol) propionic acid at 23°C. All experiments were performed in wide polypropylene D. melanogaster vials (model no. GEN32-121; Diamed Lab Supplies, Inc., Mississauga, ON, Canada). An equal number of male and female flies aged 7 to 10 days old were used for experiments unless otherwise stated.

### Assessing *p*-cresol toxicity in adult D. melanogaster.

Lethal exposure experiments were used to determine the best fly strain and toxin delivery method/concentration to model *p*-cresol toxicity. An equal number of male and female flies were used for screening experiments. Prior to the experimental start point, flies were gently anaesthetized with CO_2_ and transferred from standard rearing medium to vials containing 1% agar (wt/vol) or standard medium spiked with toxin for lethal exposure experiments. For flies on 1% agar, the capillary feeder method was used to supplement sterile ddH_2_O plus 5% sucrose (wt/vol) spiked with either 0 mg/mL, 1 mg/mL, 5 mg/mL, 10 mg/mL, or 15 mg/mL of toxin (72); those transferred to standard medium spiked with toxin received *p*-cresol at 0%, 0.5%, and 1% (wt/vol). Survival and food intake were monitored daily at least every 24 h. Any early deaths (<1 h) were assumed to be from the transfer process and were removed from subsequent analyses. We selected 10 mg/mL of *p*-cresol delivered via CAFE for the main experiments because it represents an appropriate midpoint from the initial toxicity tests.

### Oral supplementation of bacteria to D. melanogaster.

D. melanogaster *w^1118^* flies were sorted into cohorts and starved for 90 min. Flies were then transferred to standard rearing medium coated with either bifidobacteria, E. coli DH5α, or vehicle for 24 h. Bacteria were prepared for supplementation as follows: overnight cultures were washed once before being resuspended in 1 mL of sterile PBS. Then, 50 μL of the resuspension was evenly distributed to the surface of standard rearing medium and allowed to dry in a 37°C incubator anaerobically (probiotic strains) or aerobically (E. coli DH5α). Following supplementation, the flies were moved to fresh vials of sterile standard medium.

### Culture-based enumeration of *Bifidobacterium* in the D. melanogaster gut.

Ten flies (five male and five female) were surface-sterilized with 70% ethanol and then homogenized with three to five 2-mm glass beads in 500 μL of PBS using a BioSpec 3110BX Mini Beadbeater 1 (Fisher Scientific catalog no. NC0251414). Homogenates were serially diluted in PBS and plated on BSA (35). The plates were grown anaerobically at 37°C for 24 to 48 h prior to CFU enumeration. Subsequent CFU on BSA plates were counted and confirmed to be *Bifidobacterium* spp. based on morphological characteristics and Gram stain analysis.

### Assessing how bacterial supplementation alters *p-*cresol toxicity in D. melanogaster w^1118^.

The flies were sorted into cohorts and supplemented with individual strains of bacteria as described. Flies were then transferred to vials containing 1% agar (wt/vol) and fed sterile ddH_2_O plus 5% sucrose (wt/vol) spiked with 10 mg/mL *p*-cresol for 72 h. Bacterial supplementation and *p*-cresol exposure were repeated until no flies remained (Fig. 4a). Survival was recorded every 24 h after the experimental start-point.

### DNA extraction for quantitative PCR (qPCR)-based quantification of *Drosophila* gut bacteria.

After two rounds of bacterial supplementation and toxin exposure (Fig. 4a) 10 flies (5 male and 5 female) were removed from each group. Individual flies were surface-sterilized with 70% ethanol for 1 to 2 min and washed with sterile water. Flies were stored at −80°C until DNA was extracted. DNA extraction was performed as previously described (56, 73) with the Qiagen QIAamp DNA minikit (Qiagen catalog no. 51304). Briefly, single flies were homogenized in 180 μL of ATL buffer containing 20 μL of proteinase K at 56°C for 30 min. Following this incubation, flies were homogenized by bead beating at 4,800 rpm with 0.1-mm (zirconia/silica; BioSpec catalog no. 11079101z), 0.5-mm (zirconia/silica; BioSpec catalog no. 11079105z), and 1-mm (glass) beads using a BioSpec 3110BX Mini Beadbeater 1 for 3 to 5 min, followed by a second 30-min incubation at 56°C. Then, 200 μL of lysis buffer AL was added, and the samples were incubated at 70°C for 30 min and then 95°C for 10 min. The remainder of the extraction protocol was performed as per the manufacturer’s instructions.

### qPCR analysis.

The DNA isolated from D. melanogaster was used for qPCRs with the Power SYBR green kit (Applied Biosystems catalog no. 4368702) to determine bacterial loads using the universal and genus-specific 16S rRNA primers listed in Table S2. For total bacterial loads, *Dros_rt_1* (*Drosophila* actin gene) was used as the endogenous control. For quantification of specific genera, samples were normalized against the universal bacterial primer. Reagent volumes for 10-μL reactions, performed in technical triplicate, consisted of 2.5 μL of DNA, 5 μL of Power SYBR (2×), and 2.5 μL of forward and reverse primer mix (4 μM each stock). Reaction conditions were 50°C for 2 min, then 95°C for 10 min, followed by 40 cycles of 95°C for 15 s and 60°C for 1 min. qPCR was performed on a QuantStudio 5 real-time PCR system (Thermo Fisher Scientific) and analyzed using the associated QuantStudio Design and Analysis software v1.5.2 (Thermo Fisher Scientific). Relative abundance was determined by 2^Δ^*^CT^*, where Δ*CT* was determined by *CT*_Calibrator_ − *CT*_Target_. Copy numbers of target 16S rRNA genes were calculated as previously described using established primer efficiencies and limits of detection (56, 74).

10.1128/msphere.00446-22.4 TABLE S2 qPCR primers. Download Table S2, XLSX file, 0.01 MB. Copyright © 2022 Stuivenberg et al. 2022 Stuivenberg et al. https://creativecommons.org/licenses/by/4.0/ This content is distributed under the terms of the Creative Commons Attribution 4.0 International license.

### Quantifying reactive oxygen species production in *Drosophila*.

Following 2 courses of bacterial supplementation and toxin exposure (Fig. 4a), oxidative stress was assessed in the *Drosophila*. Confocal microscopy was used to visualize ROS in the Malpighian tubules that were dissected (75) and stained (76) with 2′,7′-dichlorofluorescein (Sigma) as previously described. Images were acquired using a Nikon A1R confocal microscope and the associated NIS-Elements software. Samples were exposed to white or blue light for imaging. Images were saved as .ND2 files and were imported to ImageJ (version 1.53f) for quantification.

Hydrogen peroxide content in whole flies was quantified using the Amplex Red hydrogen peroxide/peroxidase assay kit (Invitrogen catalog no. A22188) as done previously with slight adaptation (56). Four adult flies (two female, two male) were collected and homogenized in 500 μL of PBS with three 2-mm glass beads by beating at 4,200 rpm for 10s. Samples were centrifuged at 12,000 × *g* for 3 min at ambient temperature, and 50 μL of supernatant was used for the assay following the manufacturer’s instructions and spectrophotometry quantification at 560 nm with a microplate reader (BioTek Eon). Hydrogen peroxide content was normalized to total protein in the samples obtained from the H_2_O_2_ determination protocol and displayed as H_2_O_2_ relative to the vehicle. Protein quantification was done using a bicinchoninic acid (BCA) protein assay kit (Invitrogen catalog no. 23227) following the manufacturer’s instructions; 25 μL was used for each quantification reaction using a microplate reader at 562 nm.

### Statistical analyses.

All statistical comparisons were performed using GraphPad Prism 9.0 software. Data values were tested for normality using the Shapiro-Wilks test or D’Agostino and Pearson normality test. Nonparametric data were statistically compared with an unpaired Mann-Whitney test or an unpaired, one-way Kruskal-Wallis test, complemented with Dunn’s multiple-comparison test. Normally distributed data were compared with an unpaired, one-way or two-way analysis of variance (ANOVA), complemented with Dunnett’s multiple-comparison test or an unpaired, two-way Student’s *t* test.
