# Supplementary material for: Probiotic Bifidobacteria Mitigate the Deleterious Effects of para-Cresol in a Drosophila melanogaster Toxicity Model
Source: mSphere. 2022 Nov 2;7(6):e00446-22. doi: 10.1128/msphere.00446-22 (PMC9769938; doi:10.1128/msphere.00446-22)
Supplement: FIG S1 [file msphere.00446-22-s0003.pdf]

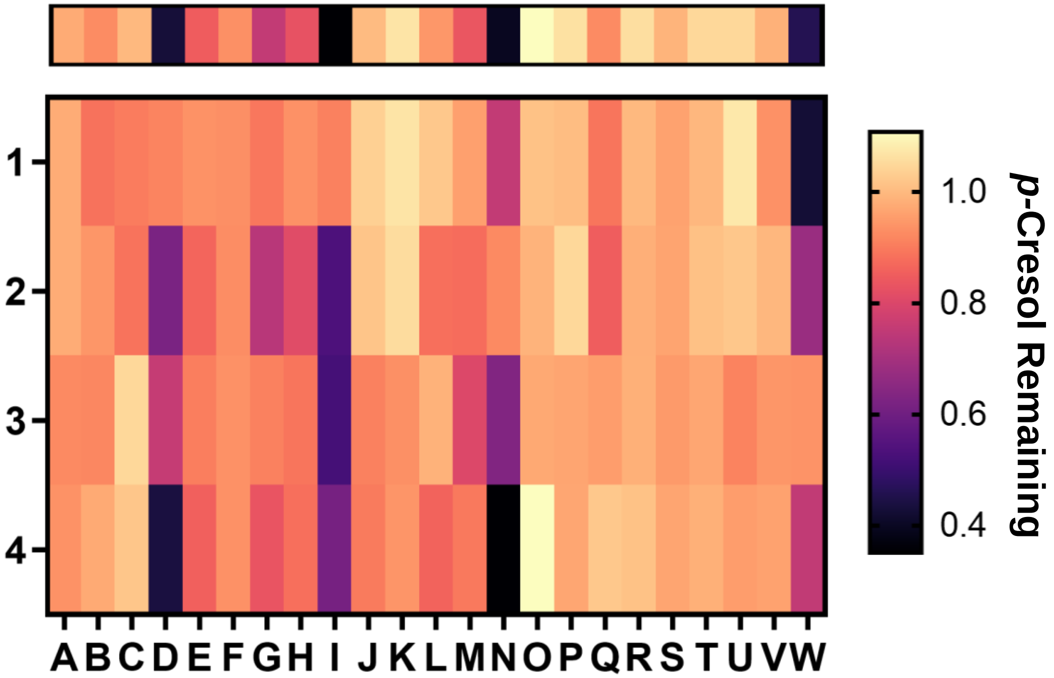

A: *B. infantis* SD-M63V-JP
B: *L. reuteri* SD-LRE2-IT
C: *L. crispatus* SD-LCR01-IT
D: *B. longum* SD-CECT7347-SP
E: *B. lactis* SD-CECT8145-SP
F: *L. rhamnosus* SD-GG-BE
G: *B. breve* SD-BR3-IT
H: *L. rhamnosus* SD-LR6-IT
I: *B. longum* SD-BB536-JP
J: *B. lactis* SD-BS5-IT
K: *L. plantarum* SD-LP1-IT
L: *L. fermentum* SD-LF8-IT
M: *B. adolescentis* SD-BA5-IT
N: *B. animalis* HRVD574-US
O: *L. casei* SD-CECT9104-SP
P: *L. plantarum* SD-LPLDL-UK
Q: *L. rhamnosus* HRVD113-US
R: *L. salivarius* SD-LS1-IT
S: *L. reuteri* RD830-FR
T: *B. lactis* SD-MB2409-IT
U: *B. longum* HRVD90b-US
V: *L. casei* HRVD300-US
W: *B. breve* HRVD521-US

SUPP FIG 1. Blind screen for *p*-cresol clearing ability in 23 lactic acid bacteria isolated from commercial probiotic product. Heat-map of *p*-cresol remaining after 24 h incubation. Data is displayed as individual replicates (bottom box) and mean (top bar) proportion of *p*-cresol remaining. Each strain was grown in minMRS with *p*-cresol at 0.2 mg/mL (n=4).
