# Supplementary material for: Probiotic Bifidobacteria Mitigate the Deleterious Effects of para-Cresol in a Drosophila melanogaster Toxicity Model
Source: mSphere. 2022 Nov 2;7(6):e00446-22. doi: 10.1128/msphere.00446-22 (PMC9769938; doi:10.1128/msphere.00446-22)
Supplement: FIG S2 [file msphere.00446-22-s0004.pdf]

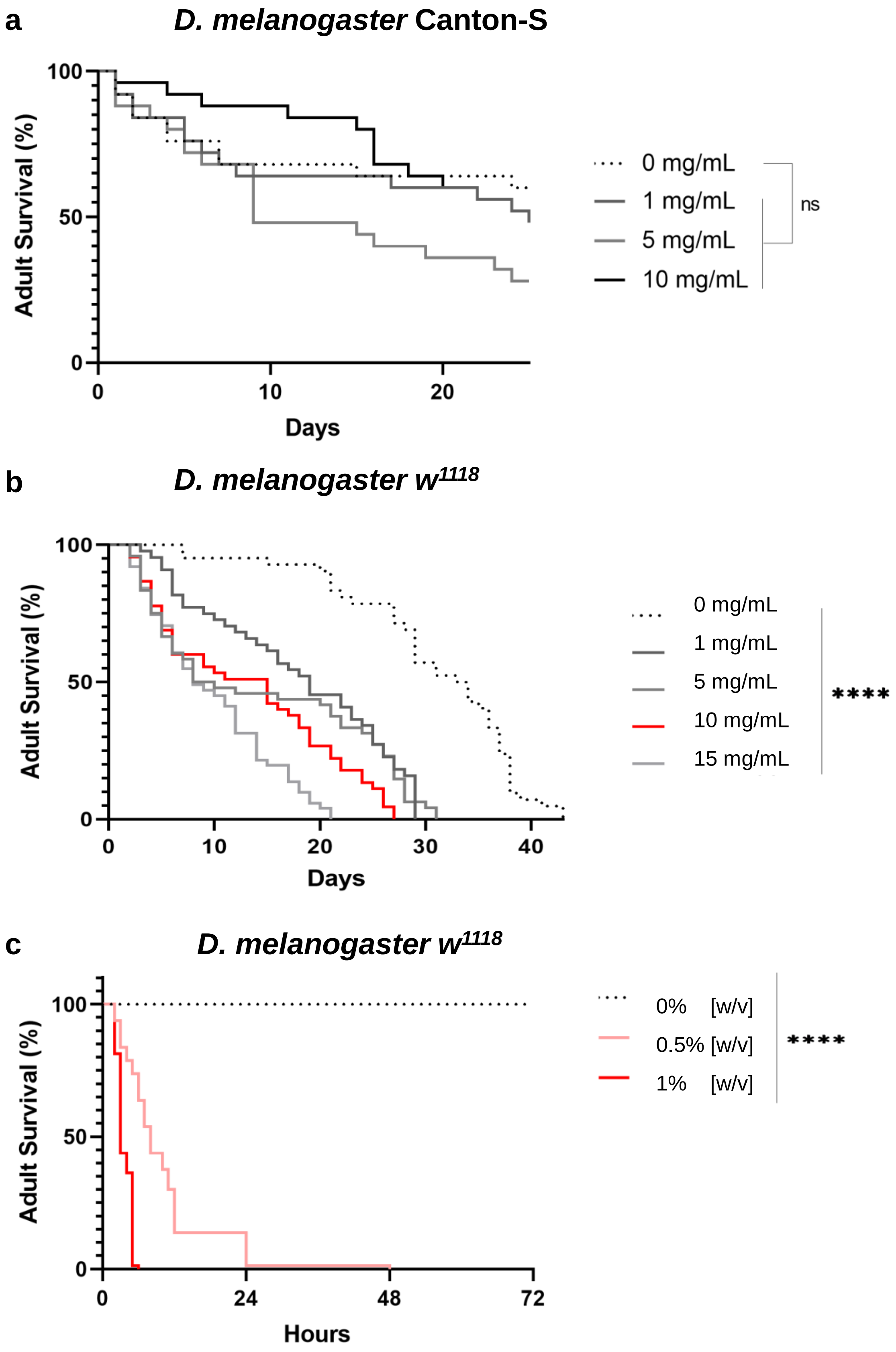

SUPP FIG 2. *p*-Cresol reduces the lifespan of *D. melanogaster* w^{1118} flies. Survival curves for (a) Canton-S or (b & c) w^{1118} flies on *p*-cresol or vehicle. Flies were supplemented *p*-cresol or vehicle using the CAFE method (a & b) or in solid media (c) (n= 20 to 30 for each group). Statistical analyses shown are from log-rank (Mantel-Cox) tests. *, $P < 0.05$; **, $P < 0.01$; ***, $P < 0.001$; ****, $P < 0.0001$; ns, not significant.
